# Validation of a Cell Proliferation Assay to Assess the Potency of a Dialyzable Leukocyte Extract Intended for Batch Release

**DOI:** 10.3390/molecules24193426

**Published:** 2019-09-20

**Authors:** Gregorio Carballo-Uicab, José E. Linares-Trejo, Gabriela Mellado-Sánchez, Carlos A. López-Morales, Marco Velasco-Velázquez, Lenin Pavón, Sergio Estrada-Parra, Sonia Mayra Pérez-Tapia, Emilio Medina-Rivero

**Affiliations:** 1 Unidad de Desarrollo e Investigación en Bioprocesos (UDIBI), Escuela Nacional de Ciencias Biológicas, Instituto Politécnico Nacional, Ciudad de Mexico 11340, Mexico; gjcarballo@ipn.mx (G.C.-U.); jelt92@gmail.com (J.E.L.-T.); gmellados@ipn.mx (G.M.-S.); carlos.lopez@udibi.com.mx (C.A.L.-M.); 2 Departamento de Farmacología y Unidad Periférica de Investigación en Biomedicina Traslacional (CMN 20 de noviembre, ISSSTE), Facultad de Medicina, Universidad Nacional Autónoma de México, Ciudad Universitaria, Ciudad de Mexico 04510, Mexico; marcovelasco@unam.mx; 3 Laboratorio de Psicoinmunología, Dirección de Investigaciones en Neurociencias del Instituto Nacional de Psiquiatría Ramón de la Fuente, Cuida de Mexico 14370, Mexico; lkuriaki@imp.edu.mx; 4 Departamento de Inmunología, Escuela Nacional de Ciencias Biológicas, Instituto Politécnico Nacional, Ciudad de Mexico 11340, Mexico; sestradap07@hotmail.com; 5 Laboratorio Nacional para Servicios Especializados de Investigación, Desarrollo e Innovación (I+D+i) para Farmacoquímicos y Biotecnológicos, LANSEIDI-FarBiotec-CONACyT, Ciudad de Mexico 11340, Mexico

**Keywords:** dialyzable leukocyte extract, Transferon^®^, complex mixture of peptides, quality specifications, biological potency, development and validation

## Abstract

Transferon^®^ is a blood product with immunomodulatory properties constituted by a complex mixture of peptides obtained from a human dialyzable leukocyte extract (DLE). Due to its complex nature, it is necessary to demonstrate batch consistency in its biological activity. Potency is the quantitative measure of biological activity and is also a quality attribute of drugs. Here we developed and validated a proliferation assay using Jurkat cells exposed to azathioprine, which is intended to determine the potency of Transferon^®^ according to international guidelines for pharmaceuticals. The assay showed a linear response (2.5 to 40 µg/mL), coefficients of variation from 0.7 to 13.6% demonstrated that the method is precise, while *r*^2^ = 0.97 between the nominal and measured values obtained from dilutional linearity showed that the method is accurate. We also demonstrated that the cell proliferation response was specific for Transferon^®^ and was not induced by its vehicle nor by other peptide complex mixtures (glatiramer acetate or hydrolyzed collagen). The bioassay validated here was used to assess the relative potency of eight released batches of Transferon^®^ with respect to a reference standard, showing consistent results. The collective information from the validation and the assessment of several batches indicate that the bioassay is suitable for the release of Transferon^®^.

## 1. Introduction

Dialyzable leukocyte extracts (DLEs) are blood products obtained from healthy human donors. These extracts are composed of complex mixtures of peptides that induce immunomodulatory activity [1,2,3]. 

Several DLEs have been used worldwide as food supplements but only a few are considered drug products after establishing several controls in the process and in the quality of the finished product. Among the controls, the consistency of batches is essential to ensure the efficacy and safety of a drug product. Consistency is demonstrated through the compliance of the criteria established in quality specifications using suitable analytical methods to assess identity, purity, heterogeneity, and potency [3,4]. 

The development of analytical methods to be included in the specifications is key to guaranteeing the robustness of the results. Therefore, these methodologies must be validated to demonstrate that the system is suitable for its intended use under a quality control environment [5,6]. However, the development of the methods employed to determine the quality attributes of a drug product containing a complex mixture of peptides is challenging due to its intrinsic heterogeneity [7]. Potency assays are critical for determining batch-to-batch biological activity. In general, in vitro potency methods require cell lines that express specific receptors that recognize the analyte and the measurement of the response elicited by the drug (for example, proliferation, death, cytokines, or growth factors expression) [6,8]. 

The properties of Transferon^®^, such as polydispersity of low-molecular-weight peptides, confirm that it is a complex drug [9]. In most of the complex drugs, the mechanism of action is not well understood; however, their therapeutic effect has been well determined for some diseases. Transferon^®^, a drug product made of DLEs, has been tested in several models. Whereas the in vitro assays allow for obtaining basic knowledge [2,10], the in vivo animal models allow for the evaluation of its effects on pathologies such as viral diseases and neoplasia [1,11]. In humans, it has been specifically proved to be effective as a coadjuvant to conventional therapies of certain diseases involving immunological dysregulation, such as major depressive disorders [12], hypersensitivities (allergic rhinitis, atopic dermatitis, allergic asthma) [13], and some infectious diseases such as herpes zoster and sepsis [14,15].

So far, the batch release tests of Transferon^®^ include an in vivo assay, which evaluates the effect of Transferon^®^ on the survival of mice infected with herpes simplex virus 1 [11], and an in vitro assay, which analyzes the ability of Transferon^®^ to induce interferon-γ expression in Jurkat cells [3]. Although the two methods are convenient alternatives used to determine the biological activity of Transferon^®^, both also present some disadvantages. For instance, the in vivo assay is affected by multiple variables, and implies complex logistics, high costs, and bioethical issues. Despite the issues involved with the in vitro assay, it is a more feasible alternative since it does not exhibit a dose–response curve because it is a limit test. Thus, new bioassays for the evaluation of DLEs potency are still required.

Recently, the capacity of DLEs to induce Jurkat cell proliferation exposed to azathioprine has been reported without describing the mechanism of action involved for this biological effect, additionally the assay lacks potency estimation with respect to a reference standard and system suitability for a linear response [16], which are required as a routine batch release assay in a quality control environment. In this work, we report the validation of a Jurkat-cells-based assay according to the recommendations for the development, validation, and analysis of bioassays of the United States Pharmacopeia (USP), the European Pharmacopoeia (Ph. Eur.), and the International Council for Harmonization of Technical Requirements for Pharmaceuticals for Human Use (ICH) [5,8,17,18,19]. Once validated, this bioassay was used to evaluate the biological potency of eight batches of Transferon^®^, as rendered in the demonstration of the consistency of this attribute.

## 2. Results and Discussion

### 2.1. Method Development

**Figure 1:**
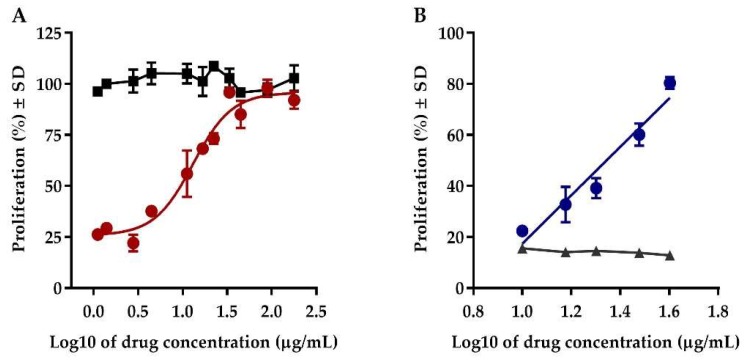
Effect of Transferon^®^ on the proliferation of Jurkat cells. (**A**) Comparison of the Transferon^®^ dose–response curve (1–180 µg/mL) using cells exposed (red circles) and not exposed to azathioprine (black squares). (**B**) Dose–response curve exhibiting a linear behavior in a concentration range from 2.5 to 40 µg/mL Transferon^®^ (blue circles) compared to the response of cells exposed to azathioprine and treated with a vehicle (grey triangles).

Proliferation inhibition of Jurkat clone E6.1 T leukemia cells is induced by a purine analogue, azathioprine, which interferes with the DNA replication [20]. Conversely, the treatment of the cells with both Transferon^®^ and azathioprine avoided this effect in a concentration range from 1 to 180 µg/mL. The obtained dose–response curve exhibited a sigmoidal behavior with a half-maximal effective concentration (EC_50_) of 13.07 µg/mL, which fitted into the four-parameters logistic model (*r*^2^ = 0.95) (Figure 1A). Based on these results we defined a concentration range of Transferon^®^ from 2.5 to 40 µg/mL to obtain a linear response (*r*^2^ = 0.94) (Figure 1B). Transferon^®^ did not modify the proliferation of Jurkat cells, unlike the inhibition observed in cells exposed to azathioprine. Additionally, the effect of Transferon^®^ was also evaluated in Daudi cells exposed to azathioprine, showing a similar dose-dependent response (data not shown). These results are evidence of a favorable effect of Transferon^®^ on cell proliferation (Figure 1). Due to the complex composition of the product, it was difficult to elucidate its mechanism of action; however, the biological responses evidenced a modulation of cytokine production, such as interleukine 6, and the ability to promote early differentiation of CD11c^+^ NK cells [2,10,11]. It allowed for the development and validation of in vitro models to contribute toward biological characterization of the product.

In our study, we confirmed that Transferon^®^ was capable of inducing the proliferation of Jurkat cells exposed to azathioprine in a specific concentration range. This was unlike the previous study, which evaluated the batch to batch consistency in biological activity using a single concentration of the product [16]. Usually, the assays for the evaluation of biologics exhibit a non-linear behavior, mainly sigmoidal. We observed this behavior; however, we established the linear range to determine the potency through parallel line analysis (PLA) [17,18,21] in order to evaluate the potency as a critical quality attribute of Transferon^®^. In this sense, the bioassay validation according to international pharmaceutical guidelines, such as ICH, USP, and Ph. Eur., is mandatory in biological potency tests as a quality specification for biologics and complex drugs [5,6,7,8,17]. 

### 2.2. Method Validation

The characteristics chosen to demonstrate that the bioassay is suitable for its intended purpose include: concentration range, precision, accuracy, specificity, and system suitability. The parameters and acceptance criteria were defined according to guidelines for pharmaceuticals and previous studies [5,8,17,21,22]. 

#### 2.2.1. Precision

Repeatability results from three independent samples for each analytic run showed percentage coefficient of variation (%CV) values lower than 25% at each concentration level of the dose–response curve. The intermediate precision (IP) values (inter-analyst, inter-instrument, and inter-assay CV) remained within the established acceptance criterion (≤25%) (Table 1). All the curves employed for the evaluation of precision showed an *r*^2^ ≥ 0.82 with no significant differences between the slopes or the intercepts obtained for each IP analysis (Table 1, Figure S1). 

#### 2.2.2. Accuracy

**Figure 2:**
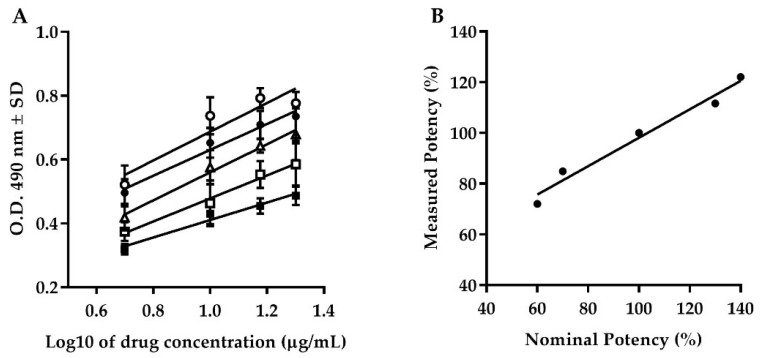
Accuracy. (**A**) Behavior of dilutional linearity at dilution levels of 60% (black squares), 70% (white squares), 100% (white triangles), 130% (black circles), and 140% (white circles) of Transferon^®^. (**B**) Relationship between nominal and measured potency at dilution levels from 60 to 140% (*r*^2^ = 0.97). O.D.: Optical Density; SD: Standard Deviation.

The results obtained from the dilutional linearity showed an upper shift in the vertical distance at 130 and 140% levels, while 60 and 70% levels showed a lower shift in the vertical distance with respect to the 100% linearity level (Figure 2A). All the curves obtained at each level of the dilutional linearity were demonstrated to be parallels using PLA analysis. All curves showed *r*^2^ ≥ 0.85 during the linear regression analysis and *r*^2^ = 0.97 between the nominal and the measured potency (Figure 2B).

#### 2.2.3. Specificity

After comparing the responses exhibited by Transferon^®^, Colagenart^®^, and Copaxone^®^, and the vehicle control, it was observed that only Transferon^®^ induced proliferation activity in cells exposed to azathioprine.

**Figure 3:**
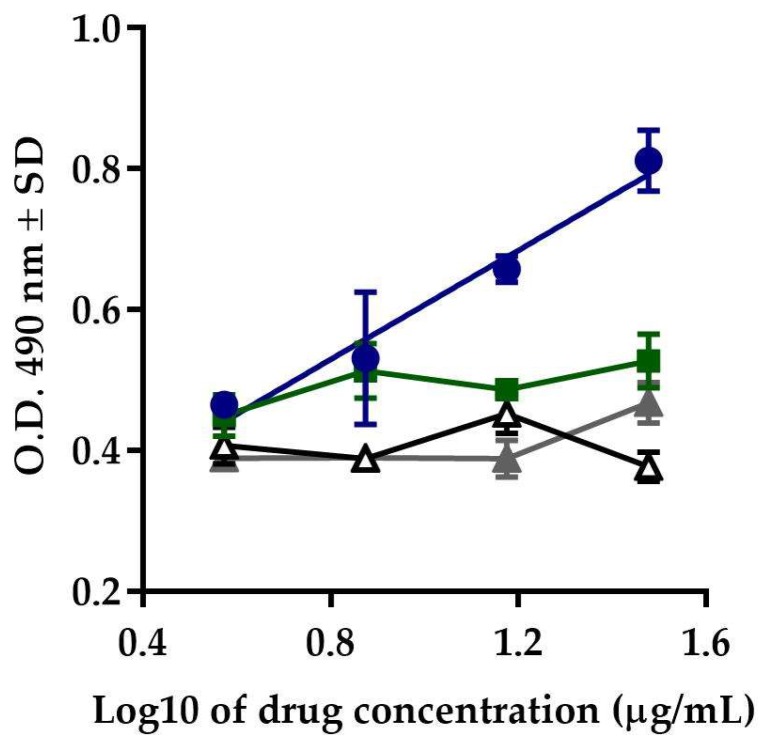
Specificity. Comparison of the response of Transferon^®^ (blue circles) with Colagenart^®^ (green squares), Copaxone^®^ (white triangles), and the vehicle control (grey triangles).

In the slope comparison analysis, Colagenart^®^ (*F*_(1, 2)_ = 3.086, *p* = 0.2210), Copaxone^®^ (*F*_(1, 2)_ = 0.023, *p* = 0.8934), and the vehicle control (*F*_(1, 2)_ = 2.836, *p* = 0.2342) did not show significant differences with respect to zero during the *F*-test. The response obtained with Transferon^®^ (*F*_(1, 2)_ = 69.06, *p* = 0.0142) remained within the acceptance criterion for this attribute (Figure 3). The results demonstrated that this method is specific for the evaluation of the biological activity of Transferon^®^. 

#### 2.2.4. System Suitability

The system suitability was established during the development and validation of the bioassay. All the established parameters were defined as pre-requisites to assess the potency of Transferon^®^ by demonstrating linearity of response, parallelism, and evident biological response (Table 2).

A linear response was observed in a concentration range (from 5 to 30 µg/mL) for all the evaluated batches (*r*^2^ ≥ 0.92) with curves constructed with five data points. The %CV was established between 0.5 to 22. All the evaluated batches showed a response ratio ≥ 2.5 compared to the control containing cells treated only with azathioprine. This means that Transferon^®^ doubled the proliferation response (optical density, O.D.) of Jurkat cells despite the presence of a cell proliferation inhibitor (azathioprine).

#### 2.2.5. Batch Consistency

**Figure 4:**
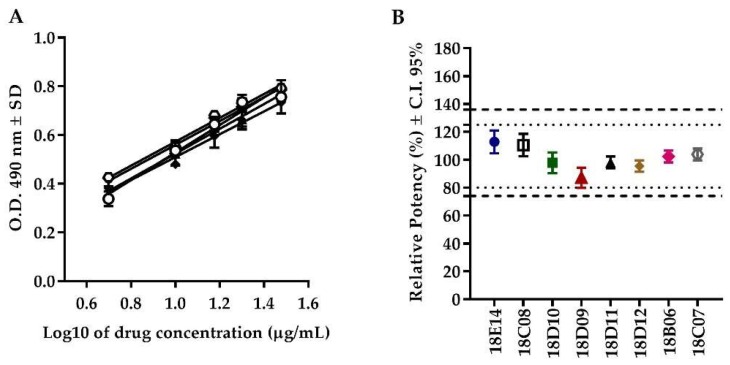
Consistency of the potency among batches of Transferon^®^. (**A**) Dose–response curves of different batches of Transferon^®^. (**B**) Relative potency of eight batches of Transferon^®^ with confidence intervals (C.I.) at 95% (*n* = 3).

The *F*-test (*F*_(4, 15)_ = 0.7519, *p* = 0.5721) showed equality of slopes in all the evaluated batches, demonstrating the parallelism among curves. The consistency of relative potency among batches was observed between 87 and 113%, while the C.I. 95% range was from 79 to 121% for the eight evaluated batches, complying with the acceptance criteria for this attribute (Figure 4 and Table 2).

The results obtained during the validation exercise showed that the bioassay was linear, precise, accurate, specific, and suitable to evaluate the biological potency of Transferon^®^. Our results demonstrated consistency in the biological potency of the product and supported our proposal of using this bioassay as an additional suitable method for batch release. Currently, the analytical methods indicated within the Transferon^®^ specification included protein content, endotoxin, sterility, identity, and biological activity [3,11]. All batches used in this work met the acceptance criteria for release using an in vivo method and the in vitro assay validated in this work. 

## 3. Materials and Methods 

### 3.1. Materials

We employed Jurkat E6.1 cells (American Type Culture Collection, ATCC, Manassas, VA, USA), Roswell Park Memorial Institute (RPMI) medium (ATCC, Manassas, VA, USA), fetal bovine serum (FBS) (Gibco, Thermo Scientific, Waltham, MA, USA), cell proliferation assay kit ([3-(4,5-dimethylthiazol-2-yl)-5-(3-carboxymethoxyphenyl)-2-(4-sulfophenyl)-2H-tetrazolium, inner salt; MTS) (Promega Corporation, Madison, WI, USA), and azathioprine (Sigma-Aldrich, St. Louis, MO, USA).

Two products containing a complex mixture of peptides were used as controls, glatiramer acetate (Copaxone^®^) acquired from Teva Pharmaceutical Industries (Central District, Israel) and hydrolyzed collagen (Colagenart^®^) acquired from LEMAR S.A.P.I. de C.V. (Mexico City, Mexico).

The development and validation of the bioassay were performed using nine commercial batches of human DLEs (Transferon^®^) (18B06, 18C07, 18C08, 18D09, 18D10, 18D11, 18D12, 18E13, and 18E14) provided by Pharma-FT (Mexico City, Mexico). Batch 18E13 was used as a reference standard.

### 3.2. Cell Culture and In Vitro Cell-Based Assay

Jurkat E6.1 cells were grown in RPMI-1640 medium with 10% FBS and placed at 37 °C in 5% CO_2_ at maximum concentration of 1 × 10^6^ cells/mL. Prior to the assay, the cells were maintained in RPMI-1640 medium without FBS for 12–18 h. Afterwards, the cells were plated at 2 × 10^4^ cells/well in a sterile 96-well plate and co-treated with azathioprine (6.25 µg/mL) and different concentrations of Transferon^®^ (2.5–40 µg/mL) in a final volume of 200 µL/well with RPMI medium supplemented with 10% FBS. Cultures were incubated at 37 °C, 5% CO_2_ for 72 h. Azathioprine-untreated cells and vehicle (water for injection) plus azathioprine were used as negative controls. After the incubation, MTS was added to every well of the plate and incubated for 4 h at 37 °C in 5% CO_2_. The O.D. was measured at 490 nm using a microplate reader (SpectraMax^®^ M3, Molecular Devices, San Jose, CA, USA, and EPOCH spectrophotometer for IP determination, BioTek, Winooski, VT, USA).

### 3.3. International Pharmaceutical Guidelines

All the work in this paper was executed following the recommendations of Chapters 1032 [8], 1033 [19], and 1034 [18] of the USP for the development, validation, and analysis of bioassays, respectively, as well as Chapter 5.3 of the Ph. Eur. [17], and Validation of analytical procedures: text and methodology Q2 (R1) from ICH [5]. 

### 3.4. Method Development

The aims of the development was, first, to evaluate whether Transferon^®^ was capable of inducing the proliferation of Jurkat cells exposed to azathioprine, and second, to establish the experimental conditions and parameters to minimize variability in the assay. In this stage, we found the concentration range that allowed for evaluating non-linear and linear responses. Additionally, we evaluated the effect of only Transferon^®^ on the proliferation of Jurkat cells as a control for the assay. The proliferation percentage (%) was calculated by dividing the O.D. of the cells treated with Transferon^®^ by the O.D. of the untreated cells with and without exposure to azathioprine.

### 3.5. Method Validation

#### 3.5.1. Precision

Precision was evaluated at the level of repeatability and IP. Repeatability was estimated through the %CV between three independent replicates during an analytic run. The IP was measured through the %CV between two analysts, two instruments, and two independent analytical runs. Five concentration levels with independent triplicates were included in all the assays. The established acceptance criterion was a CV *≤* 25% for each dilution level.

#### 3.5.2. Accuracy

Accuracy was evaluated as the dilutional linearity at dilution levels of 60, 70, 100, 130, and 140% for the dose–response curve. Relative potency was calculated with PLA analysis using the Softmax Pro 7.0.3 GxP software (Molecular Devices, San Jose, CA, USA), and the potency was relative at the 100% linearity level. The acceptance criterion for linearity was *r*^2^ ≥ 0.80 between the nominal and the measured values. 

#### 3.5.3. Specificity

Specificity was evaluated by comparing the biological response of Transferon^®^ with respect to other products composed of a complex mixture of peptides, including glatiramer acetate (Copaxone^®^) and hydrolyzed collagen (Colagenart^®^). Additionally, the obtained response was compared to the vehicle control (water for injection). The established acceptance criterion was the expected value for the characteristic biological response with Transferon^®^ while complex mixtures of peptides or the vehicle control did not show biological effect.

#### 3.5.4. System Suitability

System suitability was established according to the linear model and precision. The acceptance criteria were: linear response (*r*^2^ ≥ 0.8); ratio (Δ) between the maximum response and control response ≥ 2, using at least four points in the construction of the straight line; and CV *≤* 25%.

### 3.6. Batch-to-Batch Consitency

Relative potency was determined in eight Transferon^®^ batches using PLA analysis and an *F*-test to compare slopes. A reference standard batch was used to calculate the relative potency. The acceptance criteria were: relative potency between 80–125% and 95% confidence intervals (C.I.) between 74–136%.

### 3.7. Statistical Analysis

The fitting of raw data to the linear model and relative potency analysis were performed using the Sofmax Pro 7.0.3 GxP software. An analysis of covariance (ANCOVA) with the *F* distribution was used to test for equality of slopes. The analysis was performed using the Graph Pad Prism 6.0 software (GraphPad Software, San Diego, La Jolla CA, USA). 

## 4. Conclusions

The validation of the bioassay according to international guidelines for pharmaceutical products showed that it was suitable to evaluate the biological activity of DLEs and to determine the relative potency. In addition, we confirmed that this bioassay was also suitable to determine the relative potency among different batches of Transferon^®^, which confirmed the consistency of the quality attributes. The collective information from this study will allow for the establishment of relative potency as a critical attribute of Transferon^®^, along with the rapid implementation of this assay in a quality control laboratory as batch release analysis.

## Figures and Tables

**Table 1:** Evaluation of repeatability and intermediate precision.

Repeatability				Intermediate Precision				
	*r* ^2^	CV (%)	n	*r* ^2^	CV (%)	n	Equality Analysis	
							Slopes	Intercepts
**Analyst 1**	0.85	3.6–13.6	3	0.90	3.9–10.5	6	*F*_(1, 26)_ = 0.4851 *p* = 0.4923	*F*_(1, 27)_ = 0.3962 *p* = 0.5344
**Analyst 2**	0.95	0.7–8.9	3					
**Microplate Reader 1**	0.95	0.7–8.9	3	0.96	2.4–7.6	6	*F*_(1, 26)_ = 0.1471 *p* = 0.7040	*F*_(1, 27)_ = 0.2936 *p* = 0.5923
**Microplate reader 2**	0.96	1.9–7.5	3					
**Run 1**	0.82	7.3–22.6	3	0.83	8.4–16.6	6	*F*_(1, 26)_ = 0.3208 *p* = 0.5760	*F*_(1, 27)_ = 0.4033 *p* = 0.5308
**Run 2**	0.85	0.6–12.6	3					

**Table 2:** Results of the parameters determined during the evaluation of eight batches of Transferon^®^ for system suitability.

Batch	Potency (80–125%)	C.I. 95% (74–136%)	Δ Response (≥2)	*r*^2^ (≥0.8)	CV (≤25%)
	Results	Results	Results	Results	Results
18E13	100.0	100–100	2.9	0.96	0.7–8.9
18E14	112.8	104–121	3.5	0.94	14–22
18C08	110.5	102–118	3.5	0.92	3.0–16
18D10	97.8	90–105	2.9	0.96	3.7–14
18D09	87.0	79–94	2.5	0.95	3.3–13
18D11	98.2	94–102	3.0	0.98	4.5–9.1
18D12	95.5	91–99	2.9	0.98	1.2–7.9
18B06	102.3	98–106	3.2	0.97	0.5–2.7
18C07	103.9	99–108	3.1	0.98	0.7–4.7

## Supplementary Materials

The following are available online, Figure S1: Determination of repeatability (P) and intermediate precision (IP). (A) Inter-analysts %CV. (B) Inter-instruments %CV between different instruments (Microplate Reader MR). (C) Inter-analytical runs %CV.
