# Supplementary figures and images for: Validation of a Cell Proliferation Assay to Assess the Potency of a Dialyzable Leukocyte Extract Intended for Batch Release
Source: Molecules. 2019 Sep 20;24(19):3426. doi: 10.3390/molecules24193426 (PMC6804008; doi:10.3390/molecules24193426)

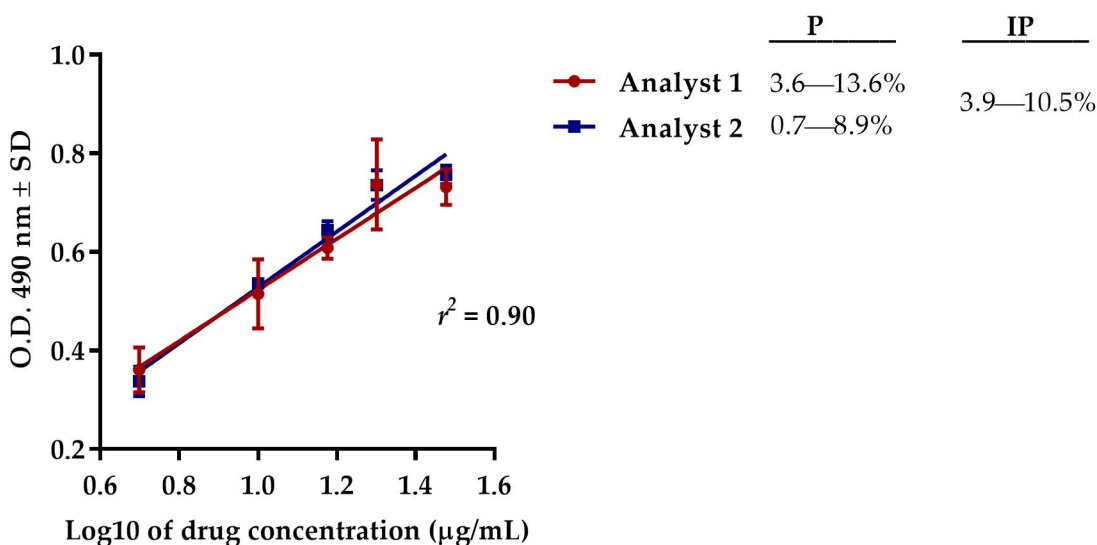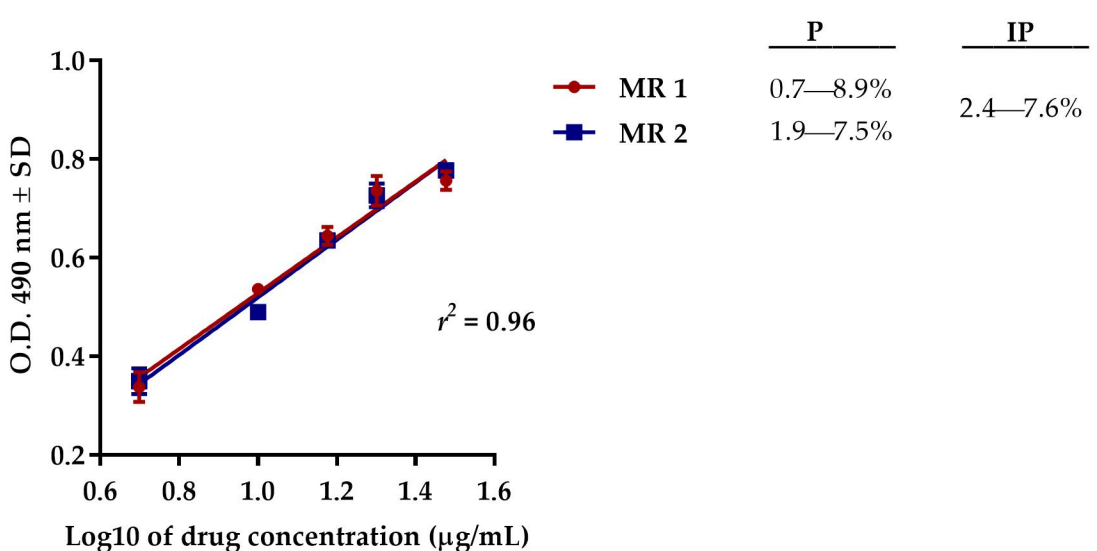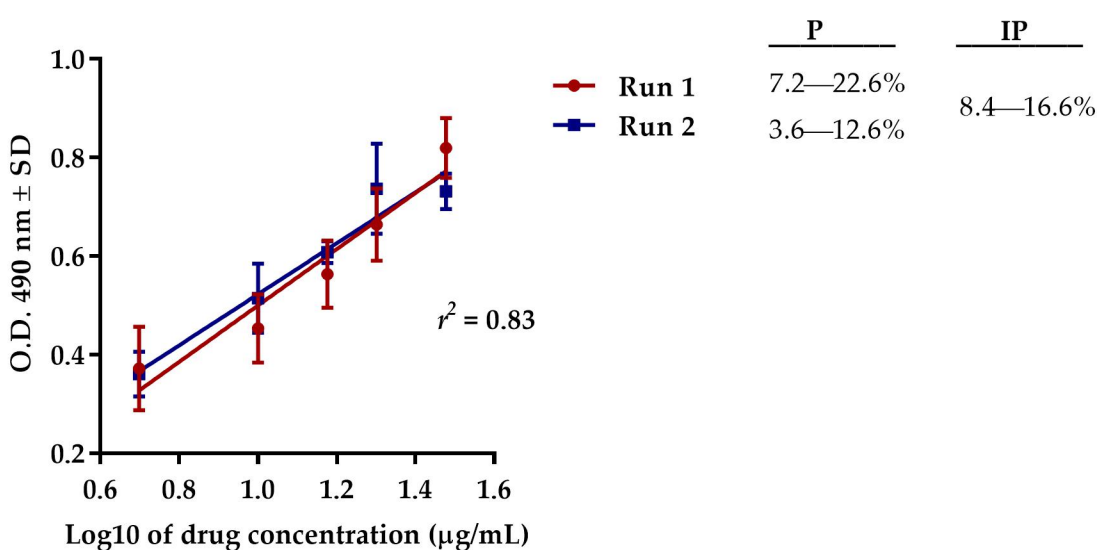

Supplement: Supplementary file 1 [file molecules-24-03426-s001.pdf]
